# Colorimetric Scale for Skin of Color: A Practical Classification Scale for the Clinical Assessment, Dermatology Management, and Forensic Evaluation of Individuals With Skin of Color

**DOI:** 10.7759/cureus.48132

**Published:** 2023-11-01

**Authors:** Philip R Cohen, Michelle A DiMarco, Rachel L Geller, Lora A Darrisaw

**Affiliations:** 1 Dermatology, University of California Davis School of Medicine, Sacramento, USA; 2 Dermatology, Touro University California College of Osteopathic Medicine, Vallejo, USA; 3 Forensic Pathology, Georgia Bureau of Investigation, Decatur, USA

**Keywords:** skin, pathology, pathologist, management, forensic, evaluation, dermatology, colorimetric, color, clinical

## Abstract

Skin of color refers to individuals whose skin color ranges from very light beige to very dark brown. Anthropologists and sociologists have previously recognized the importance of an objective classification of skin color for individuals with skin of color that does not include race and ethnicity. Since 1975, dermatologists have used the Fitzpatrick classification of sun-reactive skin types to categorize patients with skin of color; this classification was established for psoriasis patients participating in using oral methoxsalen and phototherapy clinical trial to determine the initial ultraviolet A dose. The Fitzpatrick classification merely classifies individuals as white, brown, and black; the individuals with white skin are further divided into four groups based on their burning or tanning capacity. This classification system does not provide reliable information with regard to the risk of skin cancer for individuals with darker skin color and does not aid in the evaluation of medical conditions with cutaneous involvement or assessment of appropriate cosmetic interventions for aesthetic management. Many clinicians, including forensic pathologists, incorporate the patient's race or ethnicity in their medical evaluation to describe the individual's skin color. Established scales for skin of color either include white skin color, or include 10 or more color types, or include both. We introduce a simple and rapidly performed scale that is not based on race or ethnicity to categorize persons with skin of color. The colorimetric scale ranges from very light beige to very dark brown and does not include white skin. The scale has five colors ranging from lightest (skin color type 1) to darkest (skin color type 5): very light beige (skin color type 1), light brown (skin color type 2), medium brown (skin color type 3), dark brown (skin color type 4), and very dark brown (skin color type 5); an individual with white skin would have a skin color type 0 in this classification of patient skin color. In conclusion, a scale that is not based on race or ethnicity is useful for categorizing individuals with skin of color not only for sociologists but also for clinicians who treat these patients. This colorimetric scale will be helpful for dermatologists to categorize persons with skin of color to predict their risk for developing skin cancer and to assessing appropriate cosmetic procedures and devices for these patients. In addition, the colorimetric scale will be useful for not only forensic pathologists but also other clinicians to provide a non-racial and non-ethnic designation of skin color type for their patients.

## Editorial

Introduction

Classification of skin color for individuals with skin of color is crucial for the appropriate assessment of the patient. In dermatology, skin cancer risk and treatment are influenced by the person's skin color. In addition, skin color is an essential autopsy component in the forensic evaluation of a patient.

Skin of color ranges from very light beige to very dark brown. The Fitzpatrick classification, currently used by dermatologists, only classifies individuals as white, brown, or black. Many clinicians, including forensic pathologists, currently describe the patient's skin color by incorporating the individual's race or ethnicity. A new scale to categorize persons with skin of color will be of substantial benefit for not only dermatologists and forensic pathologists but also other clinicians to provide a non-racial and non-ethnic designation of skin color type for their patients.

Discussion

The physical examination of a patient, alive or deceased, always encompasses an assessment of their skin color. White and black are not components of the visible light spectrum and therefore are not actually colors; however, not only in art but also in the assessment of skin color, they have been accepted to represent bona fide skin colors. Individuals have either "white" skin or skin of color; the latter designation applies to persons whose skin color is darker than white and ranges from very light beige to very dark brown. 

Many clinicians, including forensic pathologists, frequently described the skin color of their patients assigning the visually perceived color or the person's racial or ethnic designation or both. Currently, the Fitzpatrick classification of sun-related skin types is commonly used to designate skin color, particularly by dermatologists who evaluate patients (Table 1) [1]. The classification was introduced in 1975 and revised the following year [1].

**Table 1:** The Fitzpatrick classification of sun-reactive skin types ^a^Initially when described in 1975, the classification only included white-skinned persons. Subsequently, in 1976, brown-skinned persons and black-skinned persons were included in the classification ^b^Data from the 1975 original classification; the patients' age ranged from 12 to 40 years ^c^Data from the 1976 revised classification ^d^The erythema and tanning reactions were based on what the patient stated was their response after an initial sun exposure of three minimal erythema doses; the definition used to establish one minimal erythema dose was determined by 30 millijoules per square centimeter (mJ/cm^2^) or 15-30 minutes of noon exposure for a patient located in a northern (20-45 degrees) latitude ^e^The assessment of sunburn and tan was based on the patient's response following their first experience of moderate (three minimal erythema doses) unprotected sun exposure for 45-60 minutes ^f^All of the type 1 and type 2 patients had a pale skin color. They often had blue eyes and red scalp hair; freckling may or may not have been present. However, some patients with type 1 and type 2 sun-reactive skin had dark brown hair and blue or green eyes

Skin type^a^	Skin color of unexposed skin	Erythema reaction^b,d^	Tanning reaction^b,d^	Sunburn^c,e^	Tan^c,e^
1^b,c,f^	White^b,c^	Always burn	Never tan	Yes	No
2^b,c,f^	White^b,c^	Usually burn	Tan less than average (with difficulty)	Yes	Minimal
3^b,c^	White^b,c^	Sometimes mild burn	Tan about average	Yes	Yes
4^b,c^	White^b,c^	Rarely burn	Tan more than average (with ease)	No	Yes
5^c^	Brown^c^	Very rarely to never burns	Tans very easily	No	Yes
6^c^	Black^c^	Never burns	Always tans	No	Yes

The Fitzpatrick skin classification is initially determined by a visual assessment of whether the patient is white, brown, or black. However, for white individuals, it is further categorized based on the burning or tanning capacity. Unfortunately, this classification system does not provide reliable information with regard to the risk of skin cancer for individuals with darker skin color and does not aid in the evaluation of medical conditions with cutaneous involvement or assessment of appropriate cosmetic interventions for aesthetic management [1].

Alternative classifications for skin color classification have been incorporated by other investigators (Table 2) [1-4]. They have been used for anthropometry and racial studies [2,4]. In addition, they have been used as a component for the assessment of whether skin color influences discrimination of new immigrants to the United States [3].

**Table 2:** Comparison of color scales for individuals with skin of color CR: current report; NOC: number of categories; Ref: references

Color scale	Origin (year)	NOC	Color range	Presentation	Initial intention of the color scale	Ref
von Luschan	Late 1800s	36	White to black	36 tiles, labelled 1 to 36	To quantify skin color, based on visual matching for anthropological field research prior to the development of skin reflectance spectrophotometry (reflectometry) during the 1950s	[2]
Fitzpatrick	1975	6	White to black	Reaction to sun exposure; skin types 1 to 6	To determine the initial ultraviolet A dose for 1678 patients with generalized psoriasis participating in two trials evaluating oral methoxsalen photochemotherapy that were performed in the United States	[1]
Martin and Massey	2003	11	White to black	10 hands, extending from a white shirt; labelled 1 to 10; 0 (no hand) is the lightest skin color (such as that of an albino)	Skin color of all respondents of the new immigrant survey upon the completion of the interview was rated by the interviewer; this scale of skin color was used as a guide for investigators to determine the person's skin color	[3]
Monk	2019	10	White to black	10 circles and longitudinal bands labelled A to J	A scale to be used in social psychology and social categorization to evaluate inequalities with respect to race and ethnicity; Google subsequently partnered with Dr. Monk to use the scale to develop applications for artificial intelligence and machine learning technologies	[4]
Colorimetric	2023	5	Very light beige to very dark brown	Five colors ranging from very light beige (type 1) to very dark brown (type 5); a person with white skin would have a skin color type of 0 using this classification system of skin color	To enable clinicians who manage patients with skin of color to assess the risk of skin cancer and provide skin color-appropriate treatment guidelines for the management of medical and cosmetic skin issues and for forensic pathologists to be able to provide an objective, non-racial and non-ethnic, assessment of skin color in their autopsy evaluation of deceased individuals with skin of color	CR

The Austrian anthropologist, Felix von Luschan, in the late 1800s introduced a set of opaque-colored glass tiles; the standardized 36 tiles ranged from white to black and were used as a visual matching reference scale for recording skin color. Initially, the tiles were compared to the individual's forehead or their dorsal hand or both; subsequently, the most common site for evaluation became the inner proximal arm by the early 1920s [2].

More recently, in 2019, an associate professor of sociology at Harvard University developed the Monk skin tone scale, a 10-tone scale that ranges from white to black, to prevent people from being classified into racial categories; the benefit of this scale is that it eliminates the unintended bias that exists with the Fitzpatrick classification that excludes darker skin tones. The scale is being utilized for artificial intelligence applications and machine learning technologies by Google [4].

In 2003, a survey of new immigrants to the United States was being conducted by interviewers. The hypothesis was that people with lighter skin color fared better in a variety of social and economic settings than those with darker skin color. A scale of skin color darkness, referred to as the new immigrant skin color scale, was developed by one of the principal investigators (Douglas S. Massey) and one of the project managers (Jennifer A. Martin). The Massey-Martin scale was an 11-point scale which ranged from 0 (the lightest possible skin color, such as that of an albino) to 10; the interviewers had to memorize the scale of 10 hands, each with increased darkness, and were not permitted to refer to the scale during the interview [3].

**Figure 1:**
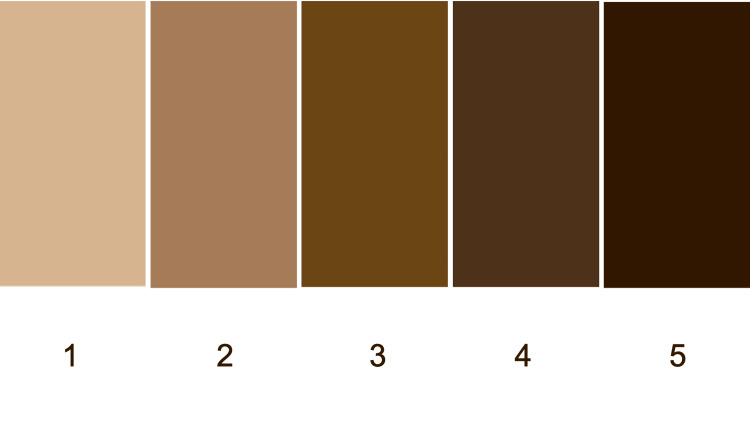
Colorimetric scale for individuals with skin of color The color ranges from very light beige to very dark brown for people with skin of color. Each color corresponds to a numerical skin color type: very light beige (skin color type 1), light brown (skin color type 2), medium brown (skin color type 3), dark brown (skin color type 4), and very dark brown (skin color type 5)

We propose a colorimetric scale for classifying skin of color (Figure 1). The scale ranges from very light beige to very dark brown since skin of color applies to persons whose skin color is darker than white. We anticipate that incorporating this colorimetric scale during the assessment of the patient will be a simple, rapidly performed visual examination by the clinician during their evaluation of the person.

The skin color type and associated color are summarized in Table 3. The colorimetric scale includes five colors ranging from lightest (skin color type 1) to darkest (skin color type 5): very light beige (skin color type 1), light brown (skin color type 2), medium brown (skin color type 3), dark brown (skin color type 4), and very dark brown (skin color type 5); white skin color is not included in this skin of color colorimetric scale. Therefore, an individual with white skin would have a skin color type 0 in this classification of patient skin color.

**Table 3:** Colorimetric classification of individuals with skin of color C: cyan (a blueish-green tint); K: key (black, used for defining details and enhancing depth); M: magenta (a purplish-red hue); Y: yellow (bright and sunny); %: percent ^a^This colorimetric classification only includes individuals with skin of color. An individual with white skin does not have skin of color. Therefore, when using this classification of patient skin color, an individual with white skin would have a skin color type 0 ^b^CMYK is a subtractive color model based on the CMY color process theory and used primarily in color printing. In CMYK, colors are produced by subtracting colors from white light. The more you add of each color, the closer it gets to black. The skin color shades can be visualized by entering the CMYK values into the CMYK calculator at the following site: https://www.w3docs.com/nx/color/color-cmyk

Skin color type^a^	Color of skin	CMYK values^b^
1	Very light beige	0%, 16%, 33%, 16%
2	Light brown	0%, 25%, 47%, 35%
3	Medium brown	0%, 36%, 81%, 58%
4	Dark brown	0%, 34%, 67%, 70%
5	Very dark brown	0%, 50%, 100%, 80%

For example, the first sentence of the clinical examination of a patient with comedones might read: a 12-year-old girl with a skin color type 1 presented for evaluation of acne. If it is an older person with white skin with a flesh-colored non-healing ulcerated facial papule, the clinical examination might begin: a 65-year-old man with skin color type 0 presented for evaluation of a suspected basal cell carcinoma. Alternatively, the first sentence of an autopsy report of a deceased patient who had been shot more than once might read: a 35-year-old man with skin color type 5 had multiple wounds created by the entry or exit of bullets.

A scale that is not based on race or ethnicity is useful for categorizing individuals with skin of color not only for sociologists but also for clinicians who treat these patients. In dermatology, a simple and rapidly performed determination to categorize persons with skin of color using a colorimetric scale can be helpful for predicting their risk for developing skin cancer and for assessing appropriate cosmetic procedures and devices for these patients [5]. In forensic pathology, a colorimetric scale for deceased individuals enables the person performing the autopsy to provide a non-racial and non-ethnic designation of skin color type for the person. 

Conclusion

In contrast to persons with white skin color, skin of color refers to individuals whose skin color ranges from very light beige to very dark brown. The importance of an objective classification of skin color for individuals with skin of color that does not include race and ethnicity has been acknowledged by anthropologists and sociologists. The Fitzpatrick classification of sun-reactive skin types is currently used by dermatologists and has been recognized to be unsatisfactory for categorizing patients with skin of color. Not only forensic pathologists but also many clinicians incorporate the patient's race or ethnicity in their medical evaluation to describe the individual's skin color. The colorimetric scale is simple and rapid to perform to categorize persons with skin of color; it is not based on race or ethnicity. The scale does not include white skin (skin color type 0) and has five colors: very light beige (skin color type 1), light brown (skin color type 2), medium brown (skin color type 3), dark brown (skin color type 4), and very dark brown (skin color type 5). In summary, the scale will enhance the ability of dermatologists to categorize persons with skin of color, allowing them to better assess skin cancer risk and appropriate aesthetic procedures and devices for these individuals. In addition, it will also enable the forensic pathologist and other clinicians to provide a non-racial and non-ethnic designation of skin color type for their patients.
